# Quantitative Complexity Theory (QCT) in Integrative Analysis of Cardiovascular Hemodynamic Response to Posture Change

**DOI:** 10.3390/life13030632

**Published:** 2023-02-24

**Authors:** Paweł Krzesiński, Jacek Marczyk, Bartosz Wolszczak, Grzegorz Gerard Gielerak, Francesco Accardi

**Affiliations:** 1 Departament of Cardiology and Internal Diseases, Military Institute of Medicine, 04-141 Warsaw, Poland; 2 Ontonix s.r.l., 22100 Como, Italy; 3 Boston Scientific S.p.A., 20134 Milano, Italy

**Keywords:** hemodynamics, impedance cardiography, syncope, cardiovascular system, complexity

## Abstract

The explanation of physiological mechanisms involved in adaptation of the cardiovascular system to intrinsic and environmental demands is crucial for both basic science and clinical research. Computational algorithms integrating multivariable data that comprehensively depict complex mechanisms of cardiovascular reactivity are currently being intensively researched. Quantitative Complexity Theory (QCT) provides quantitative and holistic information on the state of multi-functional dynamic systems. The present paper aimed to describe the application of QCT in an integrative analysis of the cardiovascular hemodynamic response to posture change. Three subjects that underwent head-up tilt testing under beat-by-beat hemodynamic monitoring (impedance cardiography) were discussed in relation to the complexity trends calculated using QCT software. Complexity has been shown to be a sensitive marker of a cardiovascular hemodynamic response to orthostatic stress and vasodilator administration, and its increase has preceded changes in standard cardiovascular parameters. Complexity profiling has provided a detailed assessment of individual hemodynamic patterns of syncope. Different stimuli and complexity settings produce results of different clinical usability.

## 1. Introduction

The assessment of hemodynamic response to different stimuli is crucial in clinical diagnosis. The explanation of the physiological mechanisms involved in the adaptation of the cardiovascular system to intrinsic and environmental demands is a domain of basic science, while clinical research has mainly aimed at identifying the cardiovascular reactions specific to particular diseases and abnormalities. 

Short-term changes in the cardiovascular hemodynamic system may be provoked by noninvasive tests, such as exercise, controlled breathing, handgrip tests, Valsalva maneuver, and posture change [1]. Novel diagnostic tools enable the continuous monitoring of cardiovascular parameters (beat-to-beat) such as heart rate (HR), blood pressure (BP), cardiac output (CO), systemic vascular resistance (SVR), and stroke volume (SV), increasing treatment effectiveness and facilitated diagnosis [2,3,4]. More advanced analysis provides their mathematic derivates, such as, e.g., heart rate variability (HRV), blood pressure variability (BPV), baroreceptor sensitivity (BRS) [5,6,7,8]. Computational algorithms integrating multivariable data have also been applied [9,10]. However, we are still far from having a “gold standard” of diagnostic and analytic methods that comprehensively depict complex mechanisms of cardiovascular reactivity. 

The Quantitative Complexity Theory (QCT) was introduced in 2005 and presents so-called “complexity science” [11]. According to this theory, complexity is no longer seen as a process but as a new physical and quantifiable property of systems. Complexity, therefore, just as, for example, energy, is an attribute of every system and may be computed based on the observable inputs and/or outputs of a given system. While conventional approaches equate complexity to entropy or to structure, the QCT combines structure—represented by the topology of the information flow between the agents in a given system—and entropy. In the QCT, the complexity function is bounded. In proximity of the lower bound, system dynamics are dominated by structure (e.g., the movement of a watch) and tends to be deterministic. In proximity of the upper bound—known as critical complexity—, it is entropy (disorder) that drives dynamics, and system behavior is stochastic (e.g., turbulent flow). The resilience of a system may be measured based on the relative values of {C_min, C, C_critical}, where C_min is the lower complexity bound, C is the current value of complexity, and C_critical is the upper complexity bound. When real-time data streaming is processed, all these values change over time. In nearly a decade, the QCT has found numerous applications in clinical scenarios [12,13,14,15,16]. According to this theory, complexity can be a new additional clinical parameter. Due to its systemic perspective, QCT can help provide the medical community with quantitative and holistic information on the state of a patient as a multi-organ dynamic system of systems. Such an approach seems adequate for assessing the overwhelming complexity of cardiovascular hemodynamic balance. 

In our previous study, we reported that QCT is a useful tool to assess the hemodynamic response of the cardiovascular system to orthostatic stress and proved it to be better than HR and BP in the predicting head-up tilt testing (HUTT) result [17]. However, our case-by-case evaluation revealed some important practical issues to be considered in the individual interpretation of the beat-to-beat complexity measurements. 

Therefore, the purpose of this paper was to reveal the nuances of the application of QCT in an integrative analysis of the cardiovascular hemodynamic response to posture change. Special attention was paid to the sensitivity of this method to dynamic changes in response to tilting, nitrate administration, and laying down, with comparisons to classic parameters, i.e., blood pressure and heart rate.

## 2. Materials and Methods

### 2.1. Participants

Three cases of athletic, non-obese healthy males are discussed in the present paper: S1 (aged 44 years), S2 (31 years), and S3 (36 years). The participants provided informed consent. The data were collected as a part of project no 126/IWSZ/2007 funded by Polish Ministry of National Defense, which was approved by the local ethics committee. The study was performed in accordance with good clinical practice standards and the 1964 Declaration of Helsinki.

### 2.2. Head-Up Tilt Testing (HUTT) 

The subjects underwent HUTT according to Italian Protocol [18] with a slight modification (passive phase 15 min). After the stabilization phase (5 min in the supine position), the subject was tilted to a position of 60–70 degrees. The passive phase of tilting was followed by a provocation phase of further 15 min after 400 of micrograms nitroglycerine (NTG) sublingual spray. Test interruption (supine restored) was made when the protocol was completed in the absence of symptoms, or when there was the occurrence of syncope/presyncope. The examination was started before 2 p.m., in fasting state, in a quiet, warm, properly ventilated, and illuminated room. 

### 2.3. Hemodynamic Assessment

Beat-to-beat hemodynamic cardiovascular response to tilting was evaluated using impedance cardiography (ICG), a noninvasive method of hemodynamic monitoring. The Niccomo™ device (Medis, Ilmenau, Germany) integrated with a Tensoscreen™ module (Medis, Ilmenau, Germany), dedicated to beat-to-beat blood pressure assessment, was used. The final analysis included several hemodynamic parameters listed in Table 1.

### 2.4. Quantitative Complexity Theory (QCT)

Complexity is a natural and physical property of every system and quantifies the amount of structured information contained therein. Conventional measures of complexity, such as Halstead complexity, cyclomatic complexity, time complexity, parametrized complexity, forecasting complexity, effective complexity, and Kolmogorov complexity—a measure of algorithmic complexity, self-dissimilarity, U-rank, or entropy, are not applicable when measuring the complexity of generic physical systems. A novel measure of complexity has been proposed [11,14] as the amount of structured information contained within a system. The complexity of a system that has a state vector {x} of N components is defined as follows: C = f(S ○ E), where E (entropy) is an N × N entropy matrix, S (structure) represents an N × N adjacency matrix, “○” is the Hadamard matrix product operator, and f is a spectral matrix norm operator. The complexity is measured in bits since entropy is measured in bits and S has no units. The above equation represents a formal definition of complexity, and it is not used in its computation. The adjacency matrix entries are 0 or 1, depending on the presence of interdependency between two state vector components. The entries of the entropy matrix are computed based on Shannon’s entropy, which constitutes the fundamental equation of Information Theory:

$$H=-\sum_{i}p_{i}\log_{2}(p_{i})$$

**Figure 1:**
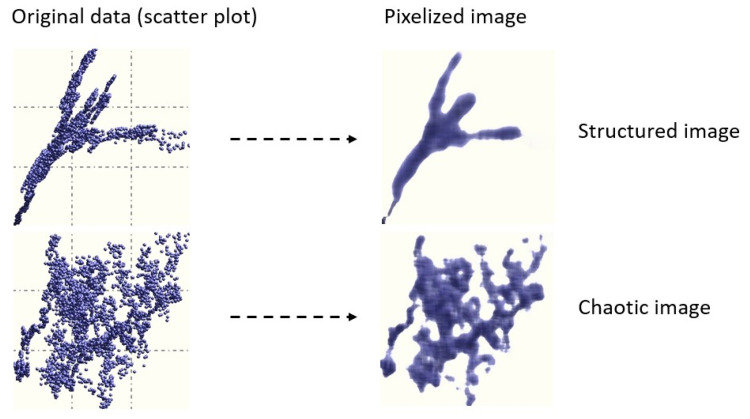
Examples of images and corresponding scatter plots [17].

The presence and intensity of interdependency between the components of {x} (so-called generalized correlation) is computed based on a proprietary algorithm which transforms scatter plots to images (Figure 1) [17]. To determine if a given image is structured (i.e., if two variables are correlated) or chaotic, images are treated using entropy based image processing techniques. Images were obtained by subdividing the area of a scatter plot into pixels. The optimal number of pixels is obtained using $\sqrt{M}$, where M is the number of data samples. If, for example, M = 100, the area of the scatter plot is subdivided into 10 × 10 mesh. The intensity of each pixel is proportional to the number of data points falling into it. Therefore, any pixel has a density equal to m/M, where m is the number of the data samples falling into it. The main advantage of this approach is that it is independent of the numerical conditioning of the data, the presence of outliers, and its ability to identify the existence of correlation structures where conventional methods fail [11]. 

**Figure 2:**
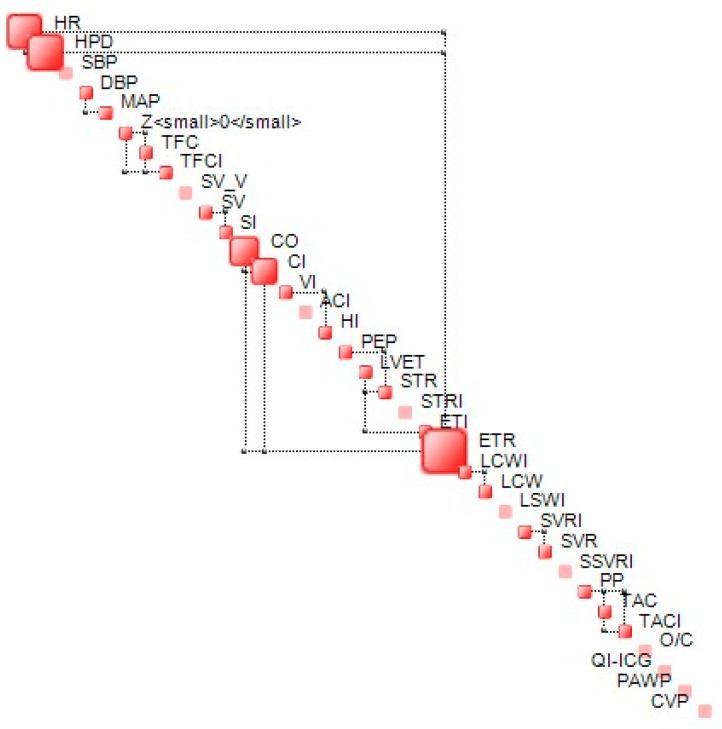
Example of complexity map. Off-diagonal connectors represent the most significant interdependencies between two variables.

The complexity metric is bounded. In proximity of the lower bound, the structural component of complexity (S) dominates the dynamics of a given system while in proximity of the upper bound—known as critical complexity—, and dynamics is dominated by uncertainty and is chaotic in nature. In proximity of the lower complexity bound, generalized correlations between the components of the state vector {x} tend to be high, while, for those close to critical complexity, these correlations tend to be weak, leading to a less stable structure. An example of a complexity map, which represents the structure of interdependencies between the components of {x} at a given time (step), is shown in Figure 2.

The software used to analyze QCT was OntoNet™ (Ontonix s.r.l., Como, Italy). OntoNet™ is a generic tool for complexity quantification, which processes data in the form of a rectangular M × N array, where M represents the number of samples, while N is the number of variables (dimensions). Data may be sampled by time, by frequency, or spatially.

## 3. Results

The descriptions of complexity evolution (window 100) during HUTT are presented in the figures and are described in detail below. Subject S1 completed the HUTT with a negative result. Two others presented vasovagal syncope type 1 (according to the Vasovagal International Study (VASIS) classification [20]), which occurred in the passive phase of HUTT in both. 

**Figure 3:**
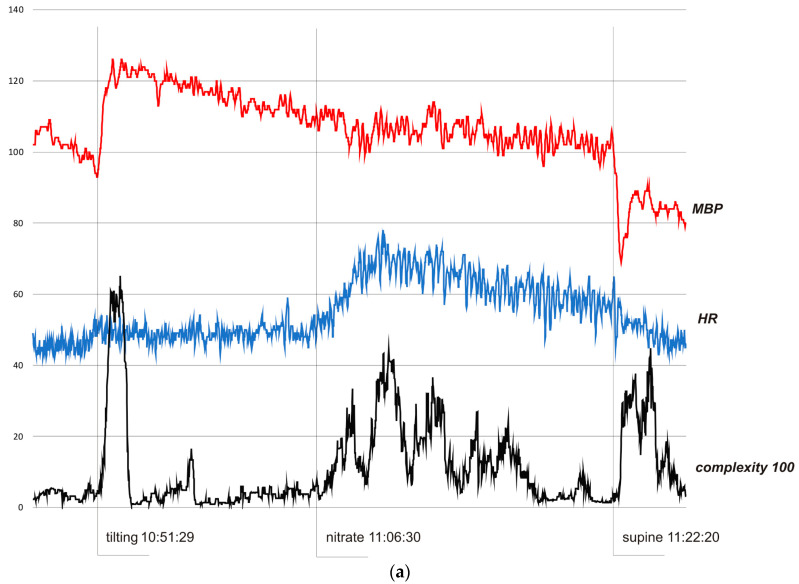

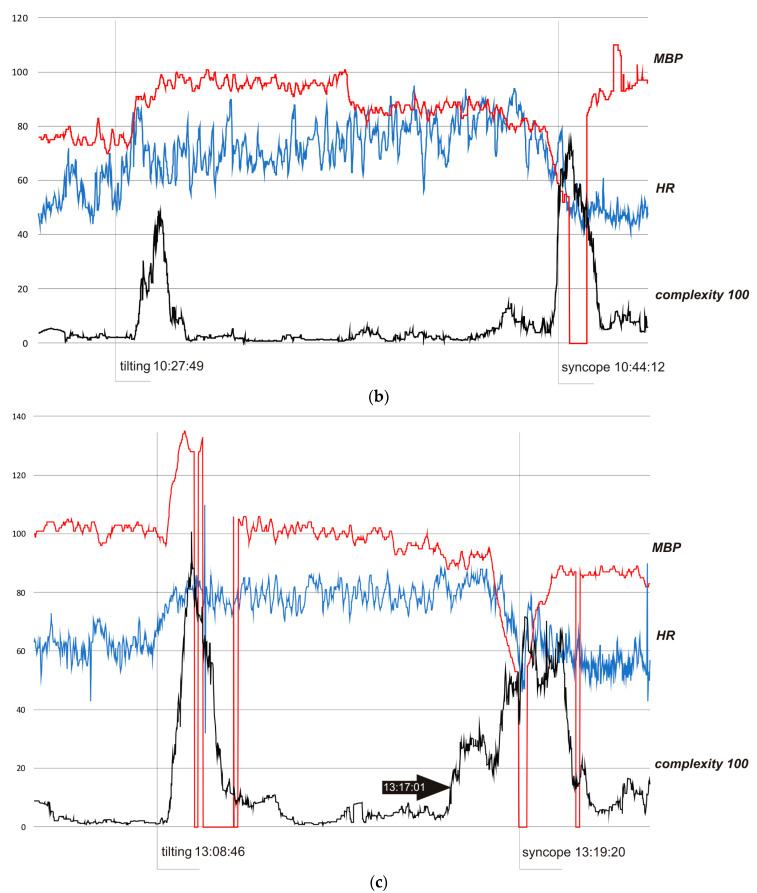
(**a**) Subject 1—trends of MBP (mmHg), HR (bpm), and complexity (beats/100). (**b**) Subject 2—trends of MBP (mmHg), HR (bpm), and complexity (beats/100). (**c**) Subject 3—trends of MBP (mmHg), HR (bpm), and complexity (beats/100).

In all cases, complexity was stable and low when the subjects were resting in the pre-tilting phase. Sudden tilting resulted in an abrupt increase in complexity followed by a decrease in baseline values after several minutes. In S1, the effect of nitrate administration strongly influenced complexity. The restoration of the supine position was also reflected by a sudden complexity peak. Figure 3b depicts a hemodynamic collapse at the end of passive phase of HUTT. The vasovagal reaction started with a progressive decrease in mean blood pressure (MBP), marked in the complexity trend by the presyncopal mound. A HR depression followed and, eventually, S2 fainted with a steep increase in complexity. In S3, a distinct increase in complexity commenced 140 seconds before syncope and preceded (by approximately 80 s) relevant changes in MBP and HR. Complexity revealed to be relatively resistant to “gaps” in MBP recordings. In all presented examples, complexity revealed to be a very sensitive and systemic marker of a complex hemodynamic response to the applied provocations.

In Figure 3a, the HUTT was negative. In the pre-tilting phase (5 min) complexity was stable (mean value 370 bits). After tilting (10:51:29), complexity suddenly rose to 6510 bits (10:53:01) and then fell to baseline value (stabilization about 10.58.00). After nitrate administration (11:06:30), it increased to 4430 bits (11:10:20) and fell to baseline after about 7.30 minutes. After supine restoration (11:22:20), complexity rose again to 4480 bits (11:24:49). In the pre-tilting phase (5 min), the mean value of HR was 45.8 bpm and the range of HR during the whole presented examination was 42–78 bpm with a maximum change from baseline 70%. MBP was 101.9 mmHg, 69–126 mmHg, and 32%, respectively. 

In Figure 3b, the HUTT was positive with syncope type 1 (mixed) according to VASIS classification [20]. In the pre-tilting phase (5 min), complexity was stable (mean value 320 bits). After tilting (10:27:49), complexity suddenly rose to 4900 bits (10:29:24) and then fell to baseline value (stabilization about 10.30). At approximately 10.40, complexity started to rise again, reaching 1290 bits at 10.42.12, then temporarily dropped to a minimum of 400 bits, and eventually rose to 7500 bits during syncope (11:44:45). In the 10.30−10.40 period, complexity was in the range of 100–500 bits (mean 240 bits). In the pre-tilting phase (5 minutes), the mean value of HR was 55.0 bpm and the range of HR during the whole presented examination was 40–95 bpm, with a maximum change from baseline of 73%. In the case of the MBP, the values were 75.4 mmHg, 52–110 mmHg, and 46%, respectively.

In Figure 3c, the HUTT was positive with syncope type 1 (mixed), according to VASIS classification [19]. In the pre-tilting phase (5 min), complexity was stable (mean value 270 bits). After tilting (13:08:46), complexity suddenly increased to 10090 bits (13:09:25) and then fell to baseline value (stabilization about 13.12:00). At approximately 13.17:00, complexity started to rise again, reaching 7170 bits during syncope (13:19:22). In the pre-tilting phase (5 min), the mean value of HR was 62.8 bpm, and the range of HR during the whole presented examination was 40–110 bpm, with a maximum change from baseline of 74%. In the case of the MBP, the values were 101.5 mmHg, 52–135 mmHg, and 49%, respectively. 

**Figure 4:**
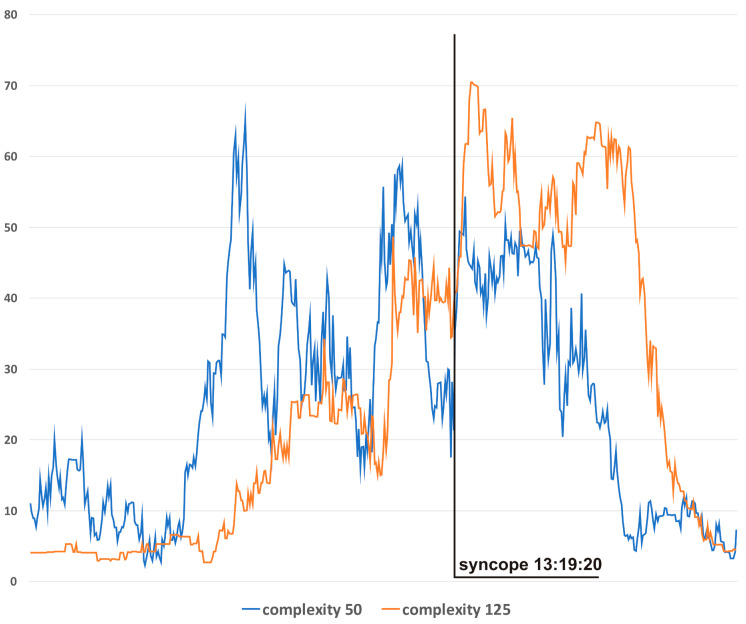
Complexity evolutions with different window sizes: blue line—window 50, orange line—window 125.

Figure 4 presents complexity trends for different window sizes for S3. In general, the narrowest window produces results with the highest variability but also with the lowest lag in relation to the actual hemodynamics. For presentation, in Figure 3c, a window of 100 was chosen as the best-suited option for this particular application.

**Figure 5:**
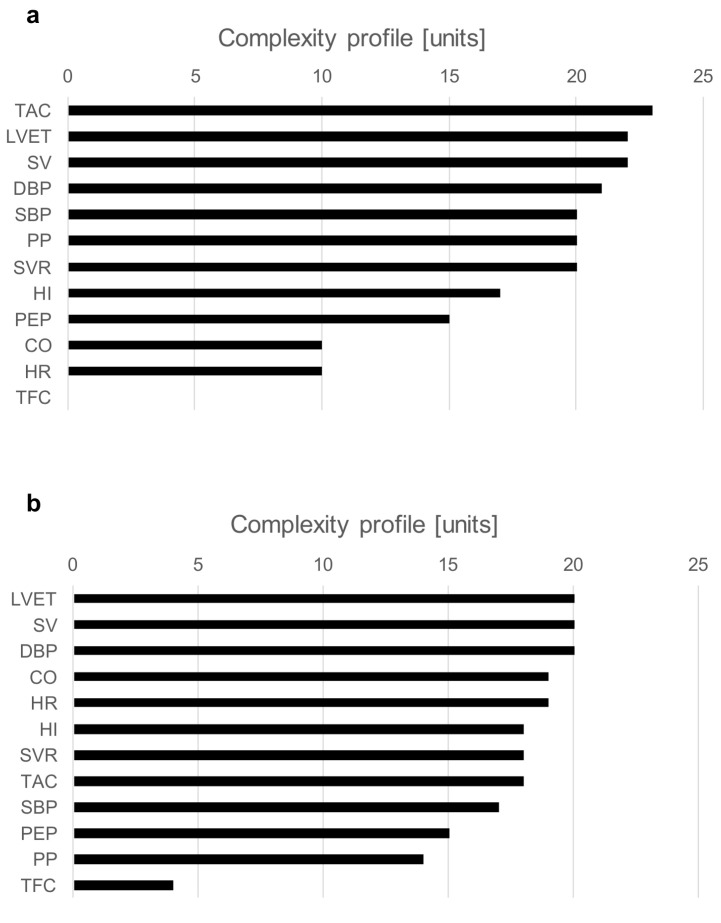
Chart (**a**)—complexity profiles at the moment of syncope event for S2; chart (**b**)—complexity profiles at the moment of syncope event for S3.

The examples of a complexity profile (CP) are presented in Figure 5a (S2) and Figure 5b (S3). The bars correspond to the contribution of each component to the total system complexity (expressed in percentages). In this example, the bar chart depicts the order of hemodynamic parameters in terms of their contribution to the vasovagal reaction. For both presented cases, SV and LVET are on the top of the complexity drivers. Although both subjects were classified as a mixed type of vasovagal syncope, there were some clear differences between them. In S2, BP indices (SBP, DBP, PP) and TAC had a greater contribution than CO and HR; meanwhile, for S3, it was the opposite.

## 4. Discussion

These clinical cases show that QCT provides a detailed assessment of the hemodynamic reaction to different stimuli. QCT reveals important details of an individual cardiovascular response to orthostatic stress and vasodilator administration. We also highlighted some pitfalls and limitations of QCT that should be considered by the users of such analytical methods. 

Various mathematical models have been developed to investigate the interaction between the complex mechanisms involved during postural changes [9,10]. In the present paper, the application of complexity derived from the QCT in HUTT was presented. Complexity, measured as a scalar function of entropy and adjacency matrices, has been shown to be a sensitive marker of cardiovascular response to the applied provocations. The integrated analysis of the representative hemodynamic parameters registered by impedance cardiography revealed strongly expressed dynamic changes in response to tilting, nitrate administration, and laying down. The complexity curve indicated decidedly more alterations from those stimuli than blood pressure and heart rate.

Resting state and stable phase of standing were reflected in low and stable complexity values. Tilting was related to an abrupt increase in complexity in all presented subjects. A similar pattern was noted after supine restoration in S1. The response to nitrate was not as dramatic: complexity increased and decreased gradually. Particularly interesting was the presyncopal change in complexity observed in S3. The early asymptomatic hemodynamic disturbances were captured more than two minutes before syncope and more than a minute before blood pressure and heart rate drop. This observation corresponds with previous reports indicating that complexity fluctuations may precede critical or life-threatening situations [12]. The value of this method in predicting cardiovascular risk was also noted. Molon et al. [15] reported that complexity and entropy indices based on QCT, calculated from 24-h beat-to-beat RR intervals in patients with heart failure and an implanted Cardiac Resynchronization Therapy (CRT) device, well represented the patients’ autonomic function and were related to a worse clinical outcome at 1-year follow-up.

We presented the prognostic value of complexity in predicting the HUTT outcome in our previous paper [17]. In 81 healthy volunteers (mean age: 37.8 years), an area under the curve (AUC) over 0.700 was observed for complexity, measured 2 minutes before the end of HUTT, with a sensitivity of 63% and specificity of 78%. This prognostic value of complexity was superior to that of the HR and MBP. Thus, we concluded that beat-to-beat complexity analysis may be used to terminate HUTT with a high probability of correct diagnosis before triggering the unpleasant symptoms of the vasovagal reflex [17].

Several earlier studies have suggested hemodynamic differences between positive and negative HUTT patients. Koźluk et al. [3] reported a higher reduction in SV (−27.2 ± 21.2 mL vs. −9.7 ± 27.2 mL; *p* = 0.03) and CO (−1.78 ± 1.62 L/min vs. −0.34 ± 2.48 L/min; *p* = 0.032) five minutes after tilting in subjects with positive HUTT results compared to HUTT-negative ones. Buszko et al. [21] identified the measures of entropy for stroke volume as the best discriminators between patients with positive and negative HUTT results. Schang et al. [22] analyzed the pre-tilting supine rest impedance waveform using neural networks and were able to predict a positive HUTT with 64% specificity and 88% sensitivity. In addition, Mereu et al. [23] derived the ratio between the RR interval and systolic blood pressure (dRR/SBP) to predict syncope 44.1 ± 6.6 s in advance with a sensitivity of 86.2% and a specificity of 89.1% (area under the ROC curve—0.877). The usefulness of machine learning emerged from the work of Hussain et al. [24], which presented a preliminary report on the effectiveness of a Support Vector Machine (SVM) in differentiating patients who do or do not have an induction of syncope and non-syncope based on their continuous and noninvasive measurements of BP and HR [24].

However, there are also studies suggesting that monitoring only one selected physiological marker may be ineffective in predicting syncope. Fu et al. [25] evaluated sympathetic neural control and vasomotor responsiveness using Muscle Sympathetic Nerve Activity (MSNA) while neutrally mediated (pre)syncope. They failed to identify a significant change in this parameter before hemodynamic collapse. MSNA decreased rapidly at presyncope, albeit much later than the initial decrease in BP. The authors concluded that it is a moderate reduction in cardiac output with coincident vasodilatation or a marked fall in cardiac output with no changes in peripheral vascular resistance that contributes to syncope.

An analysis of complexity profiles provides a deep insight into the pathophysiological background of vasovagal reaction. The charts in Figure 5a,b present differences in the individual hemodynamic mechanism within the same type of syncope (mixed). Such a detailed assessment of hemodynamic triggers may result in more effective treatment, tailored to individual patterns of vasovagal reaction.

The differences in complexity trends according to different analysis windows (Figure 4) allow researchers to choose the best option for a specific application. Narrow windows (e.g., 50) should be preferred when the early detection of hemodynamic disturbances is the priority. The advantage of a wide window (e.g., 125) lies in its higher resistance to “noise” and higher specificity to clinically relevant hemodynamic changes. 

The complexity assessment is not free from limitations. Firstly, its reliability depends on the detailed monitoring of the system. Secondly, it requires the sequential acquisition of the parameters included in the analysis, which, in many clinical settings, frequently repeated data acquisition may be challenging. Thirdly, in real life, complexity can be sensitive to many cardiovascular stimuli, which limits its specificity. The appropriate selection of the parameters for analysis may be crucial for reducing the risk of “false positive alarms”.

It is worth mentioning that other noninvasive analytic tools have also been tested to predict hemodynamics, even in the defined locations of the cardiovascular system. The computational fluid dynamic (CFD) model is an excellent example. This approach was applied for predicting the flow within the aorta and carotid arteries, helping to understand the relationship between local hemodynamics and vascular wall pathologies [26,27,28,29]. Past studies on CFDs clearly show the need for an awareness of the nuances of such advanced diagnostic tools. Morbiducci et al. [26] highlighted the importance of assumptions regarding the outflow boundary conditions. Goubergrits et al. [27] proved that the characteristics of flow at the aorta inlet can significantly affect the assessment of hemodynamics, such as the peak systole pressure gradient and wall shear stress (WSS). Moreover, Antonucci et al. [28] showed that the uncertainty of the input parameter gave a remarkable variability on the volume flow rate waveform at the systolic peak.

The cardiovascular response to upright posture depends on many mechanisms, such as the redistribution of blood volume to the lower portion of the body, vasomotor activity, a rapid fall in the central venous pressure, a marked reduction in the ventricular filling pressure, and, subsequently, a decrease in the SV. The mechanisms of adaptation are also complex: contraction of the smooth muscle of arterial and venous vessels; autonomic reflexes, resulting in an increase in heart rate, peripheral vascular resistance, venous tone, and heart contractility; the ‘skeletal muscle pump’, and neurohormones [9]. Complexity concerns many other aspects of cardiovascular function. Therefore, the undeniable advantage of QCT is its integration of different biological signals in one simple marker.

This study had some limitations. The three presented cases were of participants in a single-center study, and the analysis was of retrospectively collected data. Moreover, HUTT was performed in healthy volunteers without a spontaneous syncope history. The patterns of complexity could be different in symptomatic patients. 

## 5. Conclusions

Complexity profiling provides a detailed assessment of an individual hemodynamic pattern of syncope. Different stimuli and complexity settings (e.g., window size) produce results of different clinical usability. The users of such new analytic methods should be aware of some details and pitfalls related to the individual cardiovascular hemodynamic response to orthostatic stress and vasodilator administration. 

The possibilities of the noninvasive and continuous monitoring of cardiovascular systems rapidly develop, both in hospital settings and real life. The need for automated data analysis grows, especially with a dynamic inflow of data from the market of telemedicine m-health technologies [30]. The only way to manage this huge volume of data and to provide an adequate diagnosis and/or therapeutic recommendation is the preselection of clinically relevant incidents using advanced computed algorithms. Complexity appears to be suitable for such applications. Moreover, the analysis of complexity profiles could help to identify the leading hemodynamic abnormality, which is essential to provide adequate therapeutic intervention.

## Figures and Tables

**Table 1:** The hemodynamic parameters measured using impedance cardiography.

Parameter	Abbreviation	Comment
heart rate	HR	-
systolic blood pressure	SBP	-
diastolic blood pressure	DBP	-
mean blood pressure	MBP	-
left ventricular ejection time	LVET	-
pre-ejection period	PEP	-
stroke volume	SV	calculated as SV = VEPT * dZmax * LVET/Z0 VEPT—accounting for weight, height, and sex, Z0—baseline impedance, dZmax—maximum change of impedance (Sramek and Bernstein formula [19])
cardiac output	CO	calculated as CO = SV * HR
Heather Index		calculated as HI = dZmax * TRC TRC—the time interval between the R-peak of the electrocardiogram and C-point of impedance wave
total artery compliance	TAC	calculated as TAC = SV / pulse pressure
systemic vascular resistance	SVR	SVR = 80 * [MBP—central venous pressure]/CO

## Data Availability

The data used to support the findings of this study are available from the corresponding author upon request.
